# Supplementary material for: Catalytic mechanism of the zinc-dependent MutL endonuclease reaction
Source: Life Sci Alliance. 2023 Jul 24;6(10):e202302001. doi: 10.26508/lsa.202302001 (PMC10366529; doi:10.26508/lsa.202302001)
Supplement: Supplementary file 2 [file LSA-2023-02001_TableS1.docx]

Supplementary Table S1. Data collection and refinement statistics for the aqMutL CTDs.

| Parameters | Mn^2+^-bound form | Mn^2+^-bound form (Mn-SAD) | Zn^2+^-restored form | Zn^2+^-restored form  (Zn-SAD) | Cd^2+^-bound form of R406T | Cd^2+^-bound form of R406T (Cd-SAD) |
| --- | --- | --- | --- | --- | --- | --- |
| Data collection |  |  |  |  |  |  |
| Beamline | SPring-8 BL38B1 | SPring-8 BL38B1 | SPring-8 BL38B1 | SPring-8 BL38B1 | SPring-8 BL38B1 | SPring-8 BL38B1 |
| Detector | Rayonix MX225HE | Rayonix MX225HE | Rayonix MX225HE | Rayonix MX225HE | Rayonix MX225HE | Rayonix MX225HE |
| Wavelength (Å) | 1.0000 | 1.8920 | 1.0000 | 1.2820 | 1.0000 | 1.5000 |
| Exposure time (s) | 1 | 1 | 1 | 1 | 1 | 1 |
| Camera distance (mm) | 100 | 100 | 100 | 100 | 100 | 100 |
| Oscillation angle (∘) | 1.0 | 1.0 | 1.0 | 1.0 | 1.0 | 1.0 |
| Oscillation range (∘) | 360 | 360 | 360 | 360 | 360 | 360 |
| Space group | *P4_3_2_1_2* | *P4_3_2_1_2* | *P4_3_2_1_2* | *P4_3_2_1_2* | *P4_3_2_1_2* | *P4_3_2_1_2* |
| Cell dimensions |  |  |  |  |  |  |
| *a*, *b*, *c* (Å) | 35.6, 35.6, 167.8 | 35.6, 35.6, 167.9 | 35.5, 35.5, 167.3 | 35.5, 35.5, 167.3 | 35.6, 35.6, 167.8 | 35.6, 35.6, 167.8 |
| α, β, γ (∘) | 90.0, 90.0, 90.0 | 90.0, 90.0, 90.0 | 90.0, 90.0, 90.0 | 90.0, 90.0, 90.0 | 90.0, 90.0, 90.0 | 90.0, 90.0, 90.0 |
| Resolution (Å) | 32.80-1.22 (1.26-1.22) *^a^* | 32.76-2.30 (2.35-2.30) | 32.70-1.22 (1.26-1.22) | 34.75-1.49 (1.55-1.49) | 34.81-1.43 (1.45-1.43) | 50.0-1.83 (1.86-1.83) |
| *I*/σ*I* | 60.0 (1.1) | 53.4 (15.8) | 57.2 (4.2) | 55.5 (15.0) | 42.2 (2.6) | 51.2 (9.5) |
| CC_1/2_ (%) | 51.1 | 98.4 | 91.9 | 97.0 | 85.1 | 98.1 |
| *R*_pim_ (%) | 2.3 (57.4) | 3.9 (8.0) | 2.2 (19.6) | 2.6 (7.9) | 3.0 (32.0) | 2.1 (8.4) |
| Completeness (%) | 99.7 (99.9) | 99.3 (93.4) | 99.7 (99.2) | 98.9 (96.9) | 99.4 (99.9) | 97.1 (100) |
| Redundancy | 14.4 (13.5) | 7.2 (7.1) | 13.4 (13.6) | 5.7 (5.5) | 7.3 (7.2) | 10.7 (13.6) |
| Mosaicity (∘) | 1.26 | 0.81 | 0.36 | 0.33 | 0.69 | 0.65 |
| Refinement |  |  |  |  |  |  |
| No. of reflections | 33425 |  | 33147 |  | 20915 |  |
| *R*_work_/*R*_free_ | 0.199/0.221 |  | 0.157/0.176 |  | 0.193/0.221 |  |
| No. of atoms |  |  |  |  |  |  |
| Protein | 866 |  | 847 |  | 852 |  |
| Ligand/Ion | 13 |  | 22 |  | 12 |  |
| Water | 70 |  | 125 |  | 72 |  |
| *B*-factor |  |  |  |  |  |  |
| Protein | 22.9 |  | 17.7 |  | 20.9 |  |
| Ligand/Ion | 25.5 |  | 21.4 |  | 26.8 |  |
| Water | 28.9 |  | 24.4 |  | 30.2 |  |
| r.m.s. deviations *^b^* |  |  |  |  |  |  |
| Bond lengths (Å) | 0.006 |  | 0.006 |  | 0.013 |  |
| Bond angles (∘) | 0.89 |  | 0.88 |  | 1.26 |  |
| Ramachandran plot |  |  |  |  |  |  |
| Most favored (%) | 98.0 |  | 98.0 |  | 98.0 |  |
| Additional allowed (%) | 2.0 |  | 2.0 |  | 2.0 |  |
| Generously allowed (%) | 0 |  | 0 |  | 0 |  |
| Disallowed (%) | 0 |  | 0 |  | 0 |  |
| Protein Data Bank Code | 8H1E |  | 8H1F |  | 8H1G |  |

*^a^*Values of the highest resolution shells.

*^b^*Root mean square deviations.
