# Supplementary material for: Catalytic mechanism of the zinc-dependent MutL endonuclease reaction
Source: Life Sci Alliance. 2023 Jul 24;6(10):e202302001. doi: 10.26508/lsa.202302001 (PMC10366529; doi:10.26508/lsa.202302001)
Supplement: Supplementary file 8 [file LSA-2023-02001_TableS2.docx]

Supplementary Table S2. ICP-AES analysis of the cadmium-treated aqMutL. The wildtype or R406K mutant form of aqMutL was incubated with 0.5 mM ZnCl_2_ or CdCl_2_ at 60 ºC for 24 h. After dialysis against the metal-free buffer, concentration of zinc or cadmium was measured by ICP-AES (see Materials and Methods section).

| aqMutL | Zinc content^*1^ | Cadmium content^*1^ |
| --- | --- | --- |
| Wildtype treated with ZnCl_2_ | 4.5 | N.D. |
| Wildtype treated with CdCl_2_ | N.D.^*2^ | 2.5 |
| R406K treated with ZnCl_2_ | 4.7 | N.D. |
| R406K treated with CdCl_2_ | N.D. | 2.8 |

^*1^Number of atoms per protein molecule.

^*2^N.D. indicates that concentration of the metal was below the detection limit (0.1 μg/ml).
